# A Data Set of Synthetic Utterances for Computational Personality Analysis

**DOI:** 10.1038/s41597-024-03488-6

**Published:** 2024-06-13

**Authors:** Yair Neuman, Yochai Cohen

**Affiliations:** 1 Head, The Functor Lab, Department of Cognitive and Brain Science, Ben-Gurion University of the Negev, Beer-Sheva, 84105 Israel; 2 Gilasio coding, Tel-Aviv, Israel

**Keywords:** Human behaviour, Psychology

## Abstract

The computational analysis of human personality has mainly focused on the Big Five personality theory, and the psychodynamic approach is almost nonexistent despite its rich theoretical grounding and relevance to various tasks. Here, we provide a data set of 4972 synthetic utterances corresponding with five personality dimensions described by the psychodynamic approach: depressive, obsessive, paranoid, narcissistic, and anti-social psychopathic. The utterances have been generated through AI with a deep theoretical orientation that motivated the design of prompts for GPT-4. The dataset has been validated through 14 tests, and it may be relevant for the computational study of human personality and the design of authentic persona in digital domains, from gaming to the artistic generation of movie characters.

## Background & Summary

Human personality involves relatively stable patterns of thought, emotion, and behavior^1^. There are different approaches to reducing these patterns to a few dimensions. For instance, the most popular personality theory, the Big-Five^2^ considers human personality in terms of five dimensions such as Extraversion and Neuroticism.

Different computational tools to analyze human personality have mainly relied on this theory^3^. In contrast with the Big Five, the psychodynamic approach is the leading theoretical approach for understanding personality types in a clinical setting, as epitomized by the Psychodynamic Diagnostic Manual (PDM)^4^ and Shedler Westen Assessment Procedure (SWAP)^5^. Nevertheless, the PDM emphasizes that it aims to represent different “types” of people and that, in contrast with the DSM, it is not a taxonomy of disorders. A person can be characterized as a narcissist without having a pathology or disorder. In this paper, we use the psychodynamic approach as a theory of personality to build a dataset of utterances representing five personality types. The paper’s aim is limited to the construction and validation of a dataset, and any clinical implications are beyond the scope of the paper.

The psychodynamic approach to personality emphasizes the multidimensional structures of human personality and the role of unconscious processes and internal conflicts in shaping personality and behavior. For example, the depressive personality is presented in terms of several dimensions: The hypothetical contribution maturation patterns (e.g., genetic disposition to depression), the central tension underlying this personality type (e.g., self-criticism), the central emotion characterizing the personality (e.g., sadness), beliefs about self and others (e.g., I am a failure, and others don’t like me) and the central defense mechanisms used by the personality (e.g., devaluation of self). In other words, when characterizing the depressive personality, the psychodynamic approach describes it in terms of the main conflict underlying the personality, the emotion accompanying this tension, the representation of self and others, and the major psychological defense mechanism the person uses to cope with the stress accompanying his experience of self and others.

The assessment of personality through the psychodynamic approach is almost exclusively limited to clinical settings and the use of human expertise. The most structured tool for assessing personality through the psychodynamic approach is SWAP-200: The core of the SWAP-200, is a set of 200 items that describe various personality traits, attitudes, and behaviors. The clinicians rate these items based on their observations and interactions with the client. In other words, this tool heavily relies on human expertise and the existence of carefully selected items. Currently, no structured, automated tool can extract personality types from free text using the psychodynamic approach. We emphasize again, that the dataset presented in this paper is not a diagnostic tool for clinical settings, such as SWAP-200, but a dataset of utterances expressing five personality dimensions. The dataset is designed for researchers interested in measuring these five personality dimensions in textual data.

Given its complexity, the psychodynamic approach has seldom been used in computational personality analysis. This underrepresentation in the computational domain may result from the fact that despite its deep theoretical roots and relevance for various tasks, it lacks substantive data sets. Nevertheless, using the psychodynamic approach may be relevant to various tasks. For instance, revolutionary Large Language Models (LLMs) can imitate how different people think, feel, and behave^6^. This new technology can be used to build conversational agents that adhere to the interlocutor’s personality during a conversation. A customer characterized as highly narcissistic may be approached differently than a customer characterized as highly obsessive. Currently, we lack a substantive dataset of utterances expressing different personality types according to the psychodynamic approach, and the current paper aims to bridge this gap.

This paper provides a high-quality dataset of synthetic utterances corresponding to five personality types: depressive (DEP), obsessive-compulsive (OBS), anti-social psychopathic (PSY), paranoid (PAR), and narcissistic (NAR). We decided to focus on these five personality types for two main reasons. First, some personality types, such as anxious-avoidant and depressive, are closer to each other and cannot be easily differentiated. We decided to focus on several prototypical personality types. Second, our project is not a project in psychology but in computational sciences, and therefore, we mainly test the quality of our dataset by using machine learning classification models. Classifying utterances to an increasing number of categories (e.g., personality types) might result in a sharp decrease in the model’s performance. Therefore, we limited our current project to five personality types only.

Moreover, for each utterance, we provide a detailed description of five PDM dimensions: (1) The major psychodynamic theme expressed in the utterance (e.g., conflict with authority), (2) Affect (e.g., the expression of deep sadness), (3) Beliefs about self (e.g., I’m cable of doing almost anything), (4) Beliefs about others (e.g., People cannot be trusted), and (5) Defense mechanism (e.g., emotional detachment). This is the first dataset of its kind and may be highly relevant for a spectrum of researchers, from those dealing with computational psychology^7^ to those developing conversational agents^8–10^ and artificial personas for the gaming industry or socially assistive robots^11^. Some ways in which the data set can be used for personality R &D are:

1. Generating a digital persona for the gaming industry (e.g., generating an authentic psychopathic personality for the “bad guy”).
2. Agents for analyzing conversations of old people to identify symptoms of depression and its dynamics.
3. Automatic analysis of family therapy to provide the therapist with an online real-time analysis tool.

## Methods

We followed the approach for prompt engineering and validation tests, as successfully used in other papers^12–15^

### Generating Personas

Generating utterances expressing different personality types is far from trivial. Asking AI to generate utterances directly without specific instructions might result in caricaturists and non-representative utterances. To avoid caricaturist expressions of personality, it was highly important to us to generate a variety of imaginary characters (i.e., persona) who produce the utterances. This move was important to ensure the utterances expressing the personality type include optimal language variability. Therefore, the first step in the process was to produce different *personas, which* are the imaginary subjects generating the utterances. To address this challenge, we selected several general dimensions of the persona generating the utterance, such as gender, profession, and age. The selected dimensions are hypothesized to produce different expressions of the same personality. For example, a 35-year-old male who is the CEO of a high-tech company may express a narcissistic personality in a different way than a 90-year-old grandmother.

To address this challenge, we used GPT-4^16^ (API version GPT-4-0613) and the following prompt:

[Generating artificial persona]

Generate 100 unique American English speakers, each represented by a distinct combination of the following eight dimensions: gender, age (16-80), cultural identity, socioeconomic status, education level, family status, profession, and personal identity.

Ensure diversity across these dimensions.

##

Provide the output in the format of

Gender:

Age:

Cultural identity:

Socioeconomic status:

Education level:

Family status:

Profession:

Personal identify:

##

**Example 1:**

Gender: Female

Age: 32

Socio-economic status: Middle-Class

Education level: Bachelor’s Degree

Family status: Married with two children

Profession: Software Engineer

Cultural identity: Indian American

Personal identity: Feminist and Tech Enthusiast

**Example 2:**

Gender: Male

Age: 45

Socio-economic status: Working Class

Education level: High School Graduate

Family status: Divorced with one child

Profession: Electrician

Cultural identity: Irish American

Personal identity: Single Parent and Sports Enthusiast

**Example 3:**

Gender: Female

Age: 78

Socio-economic status: low

Education level: uneducated

Family status: Married, mother to 7, and grandmother of 8

Profession: Housewife

Cultural identity: Jewish Hasidic

Personal identity: Wife, mother, and grandmother

The outcome of this procedure is a list of 100 personas available in the data link.

### Generating the dataset

In the second phase, we used the list of personas and the following prompt’s template to generate utterances expressing different personality types. Here, we present the general template:

[Generating synthetic utterances expressing PERSONALITY TYPE]

Summary statement: [In this section, we inserted a short description of the personality type]

(In this section, we inserted a more detailed description of the PERSONALITY TYPE).

##

Your task is to generate five distinct utterances in which the PERSONA expresses a [PERSONALITY TYPES HERE] personality. Ensure that the language used accurately reflects the PERSONA’s:

- Cultural background
- Social status
- Dialect
- Interests
- Slang
- Common grammatical structures
- Jargon, and
- Unique conversational tone.

##

Here is a description of the PERSONA: [here we insert the persona’s description].

We ran the prompt on each of the 100 artificial personas. The result is a list of 5000 utterances representing the five abovementioned personality types. After completing all processes mentioned in the paper, we removed duplications, and the final file includes 4972 utterances. Each utterance is labeled with one of the five personality types/labels.

Here are three examples of utterances generated for the following persona: • Female, 84 • Low class, high school education • Widow, farmer’s wife • Afro-American housewife.

### Narcissist

“Honey, back in the day, folks just couldn’t keep up with my style. I was turning heads at the local dances, and you could tell I was the cream of the crop. Now, I don’t blame them for trying to imitate my fashion sense. It’s hard being a trendsetter, especially in this small town.”

### Paranoid

“I’ve been around the block a few times, seen folks with sly smiles and hidden agendas. Can’t trust anyone these days. They act all friendly, but I know they’re talking behind my back, plotting something. I’ve got my eyes peeled, ain’t falling for their tricks.”

### Anti-social psychopathic

“You know, them young folks come ‘round here, thinkin’ I’m just a sweet ol’ granny. Little do they know, I’m always lookin’ for a way to get what I want. Last week, the handyman tried to charge me double for fixin’ the porch. Well, I made sure he left with an empty pocket, and my porch is as good as new.”

## Data Records

The Data Records^17^ includes several files. All files are CSV files with a very simple structure. The files are (1) A list of the 100 personas (2) The file with the personality types, utterances, and accompanying data. The link also includes the code for running the SetFit model.

## Technical Validation

The technical validation section includes several validation tests to support the dataset’s quality. The tests are organized in the following clusters:

1. The judgment of human annotators,
2. Validation of the utterances through computational tools,
3. Validation through machine-learning models, and
4. validation through various datasets and indirect measures.

Each of these clusters is explained in the following sections.

### Validation 1. Validation through human experts

If the synthetic utterances validly represent the personality types underlying them, then there should be an agreement between the label of the utterance and the decision of expert human annotators.

We selected 100 utterances, 20 representing each personality type. The representative utterances for each personality type were randomly selected from those that scored highest on personality type (see validation 2) and lowest on the others. Next, we used two independent experts/annotators to identify which personality type best describes the utterance. The annotators (“A” and “N”) were two graduate students who completed a one-year personality analysis and profiling course. The course involved specific training in analyzing textual data, and “A” and “N” completed it with top grades. Each annotator received the 100 utterances presented in a random order and with the following instructions:

Here are five personality types:

1. DEPRESSIVE (DEP).
2. ANTI-SOCIAL PSYCHOPATHIC (PSY).
3. PARANOID (PAR).
4. NARCISSISTIC (NAR).
5. OBSESSIONAL (OBS).

In the attached Excel spreadsheet, you will find 100 utterances. Each utterance expresses one of the five personality types. For each utterance, describe which personality type best describes it. Write the name of the personality type using the abbreviations DEP, PSY, PAR, NAR, and OBS.

The experts’ annotations were tested against the “gold standard, “ which is the personality type (i.e., label) used to generate the utterance, and against each other.

The association between the experts’ annotations was statistically significant (Χ^2^ = 360.25, p < 0.001), representing almost a perfect agreement between them. They agreed on 95% of the cases. The association between each annotator and the gold standard was found to be statistically significant for “A” (Χ^2^ = 386.32, p < 0.001) and “N” (Χ^2^ = 369.31, p < 0.001). These results validate the quality of the utterances and the extent to which they represent personality types.

### Validation 2. Validation through computational tools

Suppose the utterances validly represent the personality type. In that case, there should be an agreement between the label of the utterance and the label given to the utterance by different computational tools. To test this hypothesis of convergent validity, we used two tests with LangChain and GPT-4.

Test 2.1

The first tool we used is LangChain^18^, which is “a framework for developing applications powered by language models. It enables applications that (1) Are context-aware: connect a language model to sources of context (prompt instructions, few shot examples, content to ground its response in, etc.), and (2) Reason: rely on a language model to reason (about how to answer based on provided context, what actions to take, etc.)”. We used LangChain to generate a dialogic and step-by-step assessment of each utterance. See the following prompt:

Prompt

User: I gave AI the task of generating synthetic utterances expressing the [PERSONALITY TYPE HERE] personality.

Here is a generated utterance: “[UTTERANCE HERE].”

Please provide your general assessment of the utterance generated by the AI. Does it authentically express the [PERSONALITY TYPE HERE] personality according to the Psychodynamic Diagnostic Manual (PDM) and SWAP?

Provide a straightforward answer in terms of YES or NO.

ChatGPT Response: [RESPONSE HERE]

**

Now, evaluate the utterance in terms of the PDM dimensions of personality: major theme or preoccupation, affect, beliefs about self, beliefs about others, and the major defense mechanism. Generate the title of the dimension followed by your assessment.

**

ChatGPT Response.

**

User: Provide a numerical rating on a scale from 1 to 5, with 1 indicating ‘not at all’ and 5 indicating ‘very much’ regarding expressing [PERSONALITY TYPE HERE] personality traits in the given utterance.

ChatGPT: A single numerical rating.

Using this procedure, we generated several additional columns to our file. First, whether the AI agrees that the synthetic utterances we evaluate express the personality type they are supposed to represent (YES or NO). Second, the degree to which the utterance expresses the personality type it is supposed to express (1-5).

If the synthetic utterances are valid, then we should expect an agreement between the original label and the label produced by LangChain. The results support the hypothesis: The LangChain outcome agreed that the utterances represent the personality types for DEP in 97% of the cases, for NAR in 98% of the cases, for OBS in 63% of the cases, for PAR in 99% of the cases, and for PSY in 65% of the cases.

Test 2.2.

Although we used GPT-4 to generate the synthetic utterances, it is unclear whether GPT-4 would label these utterances as expressing the personality type according to the label. Suppose the synthetic utterances validly represent the five personality types. In that case, there should be an agreement between the label and the label provided by GPT-4 when asked to judge which of the five personality types is expressed by an utterance. To test this hypothesis, we used the following prompt:

Based on the PDM (Psychodynamic Diagnostic Manual) and SWAP (Shedler Westen Assessment Procedure), identify the < PERSONALITY TYPE > in the following statement: “UTTERANCE HERE.”

Score each personality type on a 5-point Likert scale to represent your trust in the diagnosis.

< PERSONALITY TYPE > : < depressive, paranoid, narcissist, obsessive-compulsive, anti-social psychopathic >

## Example:

Statement: “I just pushed someone on the street and laughed when he fell on his face.”

Output:

Depressive: 1

anti-social psychopathic: 5

paranoid: 2

narcissist: 2

obsessive compulsive: 1

The outcome of this procedure was five personality scores generated for each utterance. We used this outcome for two tests.

The personality diagnosis taxonomy of SWAP^19^ suggests that paranoid, anti-social psychopathic, and narcissistic personalities are personality types belonging to the *externalizing* spectrum. In contrast, the depressive personality belongs to the *internalizing* spectrum, and the obsessional personality belongs to the *neurotic* style.

**Fig. 1:**
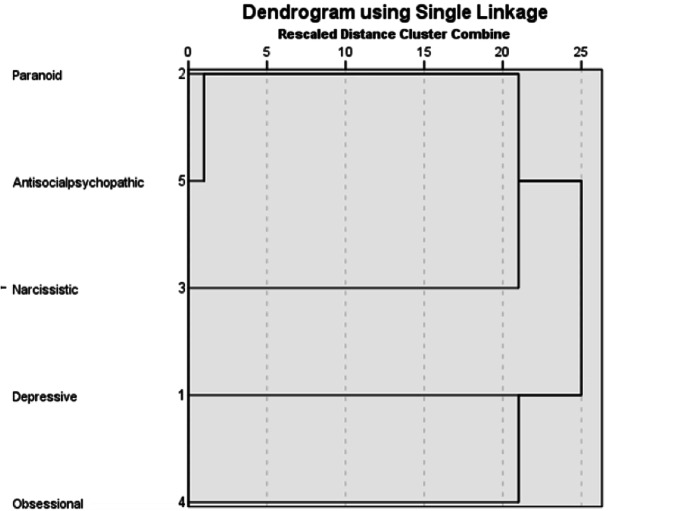
Dendrogram for the five personality measurements.

Following this theorization, we first hypothesized that if the synthetic utterances are valid, we should see a cluster of the measurements produced by GPT-4 corresponding with the taxonomy mentioned above. To test this hypothesis, we applied Hierarchical cluster analysis using IBM SPSS Statistics with the Nearest neighbors as a clustering method and Euclidean distance as a measure. In other words, we used the scores generated by GPT-4 and clustered the results. Figure 1 presents the Dendrogram.

We can see that the hierarchy corresponds to the taxonomy suggested by SWAP. This result supports the construct validity of the synthetic utterances.

Next, we tested the hypothesis that if the utterances validly represent the personality types, then, on average, there should be a match between the score given by GPT-4 and the label of the utterance. For instance, a synthetic utterance that should express depressivity should also get a higher score on depressivity than on narcissism, anti-social psychopath, obsessive, or paranoid. The mean average score for each personality type appears in Table 1:

**Table 1:** Mean averages for each personality type.

TYPE	SCORES				
	PSY	DEP	NAR	OBS	PAR
PSY	3.56	1.09	3.66	1.65	2.06
DEP	0.87	4.71	1.35	2.19	1.19
NAR	1.28	1.02	4.78	1.85	1.54
OBS	1.07	1.28	1.86	4.41	1.62
PAR	1.19	1.31	2.15	1.94	4.72

We can see that the highest score given by GPT-4 to an utterance was almost always given to the corresponding personality type. For instance, synthetic utterances labeled DEP scored highest on the depressive score given by GPT. Utterances labeled PSY scored highest on NAR and PSY, which aligns with the correlation between these two dimensions.

### Validation 3. Validation through machine learning models

We hypothesized that if the utterances validly represent the different personalities, they can be used for training and testing machine-learning classifiers.

The reason for this hypothesis is as follows. A machine-learning model is trained on a data set to identify a pattern. In our case, the pattern involves different personality types expressed by the utterance. The model’s success is tested by the ability to classify new utterances according to the patterns learned in the learning phase. If the utterances are of high quality, then we can expect the model to learn the pattern successfully,

We used three machine-learning models to test the hypothesis: CRT, SetFit, and RoBERTa.

Test 3.1

We used the five scores generated for each utterance by GPT-4 as predictive features in a machine-learning model. We hypothesized that using the five scores in a predictive model should result in good classification results if the utterances are valid. Using the Classification and Regression Tree (CRT) with ten-fold cross-validation, we predicted the personality type based on the five scores. The confusion matrix is presented in Table 2:

**Table 2:** Confusion matrix for the CRT.

	PSY	DEP	NAR	OBS	PAR	% CORRECT
PSY	785	5	121	67	22	79
DEP	0	955	3	40	2	96
NAR	42	2	890	59	7	89
OBS	16	12	50	885	37	89
PAR	34	10	25	12	911	92

We can see that the classifier trained on the five scores successfully identified the different personality types of the utterances. The percentage of correct identification (i.e., recall rates) is higher than expected by a random guess determined by the baseline (i.e., 20%). For instance, the classifier correctly identified 79% of the utterances labeled as PSY, a 59% improvement over a random guess.

Test 3.2.

Next, we selected the 15 top-ranked utterances for each personality type. To identify the top-ranked utterances, we calculated new scores for PAR, NAR, and PSY:

PAR = PARA-ANT

PSY = PSY-NAR

NAR = NAR-ANTI

as these personality types are the most similar to each other according to the psychodynamic approach. Next, we used the top 15 utterances for each personality type to train a SetFit^20^ model. If a small number of utterances that best represent a personality type validly represent it, then even these small number of utterances (i.e., 15 for a personality type) can be used to train a classifier.

The model has been tested on the rest of the utterances. The SetFit model, trained on 75 utterances only, gained 80% accuracy, precision, and recall. The performance measures for each personality type are 87% (paranoid). 68% (anti-social psychopath), 91% (depressive), 84% (narcissist), and 73% (obsessional). The code for the model is available for the researchers (see code availability section). These results further support to the quality of the utterances.

Test 3.3.

Finally, we used RoBERTa^21^ model with a ten-fold cross-validation procedure. 70% of our dataset of utterances was used to train the model, and the rest was used for the test. The baseline for prediction is 20%, as we have five personality types equally represented in the dataset. The classifier gained an average of 65% accuracy, 71% precision, and 65% recall. These performance measures present a significant improvement over the baseline and further support the quality of the utterances.

### Validation 4. Extending the ecological validity of the utterances

We conducted several indirect tests to test the ecological validity of the utterances.

Test 4.1.

We used the SetFit model to analyze the EmpatheticDialogues dataset^22^, a large-scale multi-turn dialogue dataset with utterances and their emotion. First, we focused on utterances labeled as Proud and Sad. We selected the 667 utterances labeled as “sad” and 686 as “proud.” Next, we used the previously trained SetFit model to analyze the Proud and Sad utterances. We hypothesized that Proud utterances should express a higher level of narcissistic personality than Sad utterances and that Sad utterances should express a higher level of depressive personality than Proud utterances. If this hypothesis is empirically grounded, then the Narcissistic personality score given by the SetFit model for Proud utterances would be higher than for Sad utterances and vice versa for the Depressive personality score. Confirming these hypotheses may further support the quality of the utterances, as we expect the model trained on quality utterances to be useful for identifying the expression of personality types in other contexts.

**Fig. 2:**
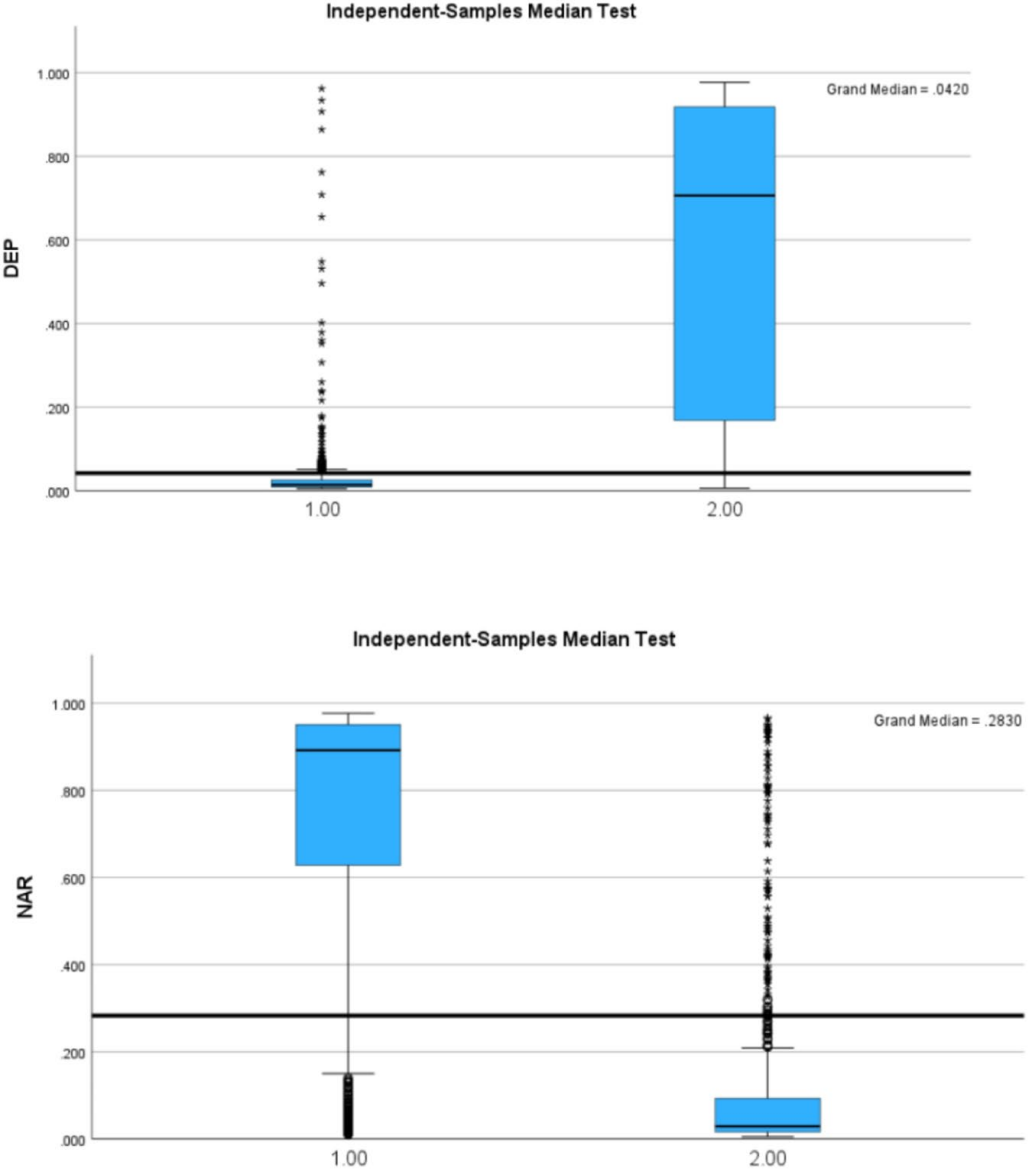
(**a,b**) DEP and NAR scores for utterances labeled as proud (1) and sad (2).

The results appear in Fig. 2a-b:

We can see that the scores support our hypotheses. The Median test was statistically significant (p < 0.001). Moreover, using the Mann-Whitney U Test, we found that the Mean Rank of Depressive personality score given to “sad” utterances was higher than the Narcissistic personality score (984.47 vs. 378.04 respectively) and that for proud utterances, the Mean Rank of the narcissistic personality score was higher (958.53 vs. 387.45 respectively). The results support the hypotheses and further validate the quality of the utterances.

Test 4.2.

Based on the emotion associated with each personality type according to the PDM, we hypothesized that if the utterances validly represent the personality types, synthetic utterances expressing depressivity should score higher on negative sentiment, and synthetic utterances expressing narcissism should score higher on positive sentiment. We tested this hypothesis.

**Fig. 3:**
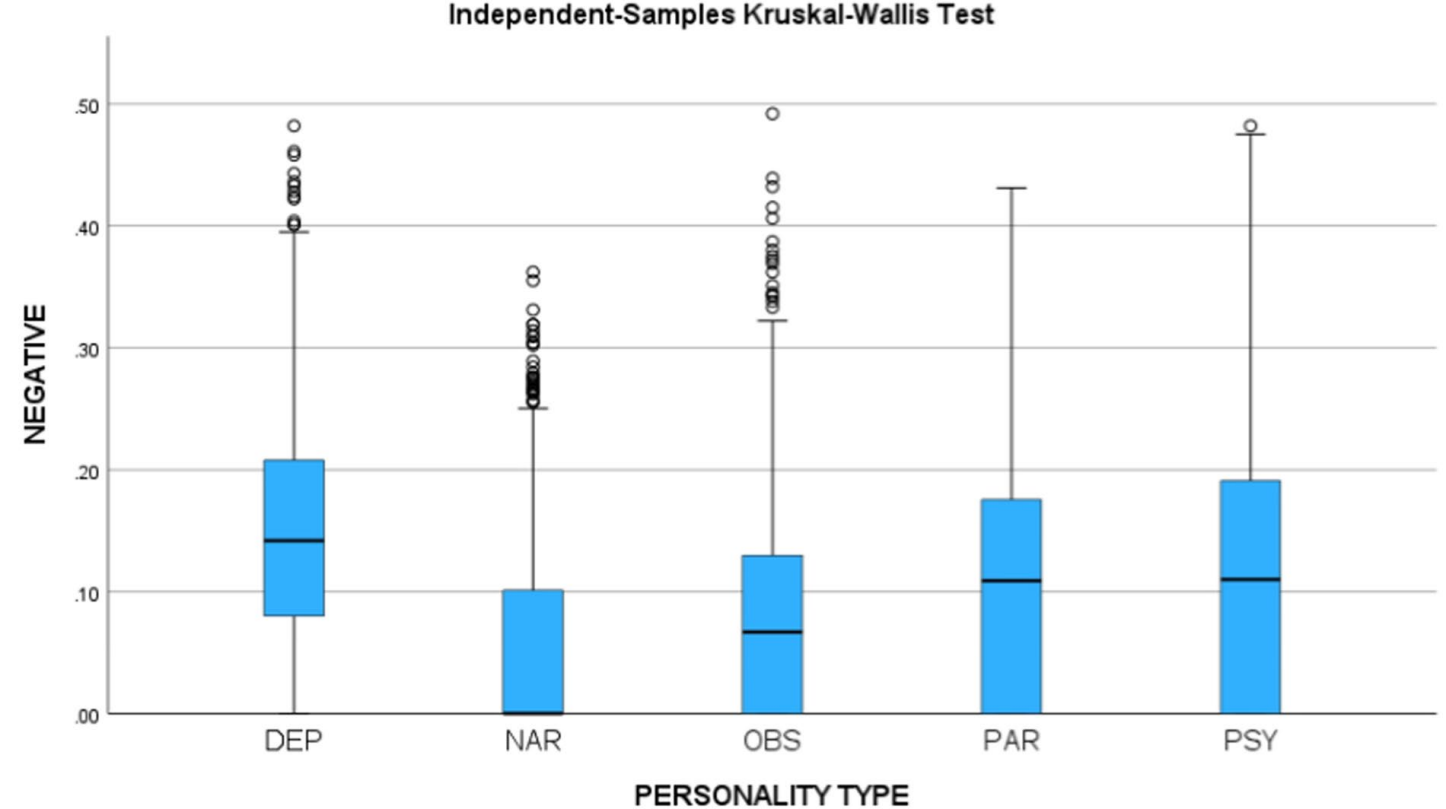
Negative sentiment scores.

**Fig. 4:**
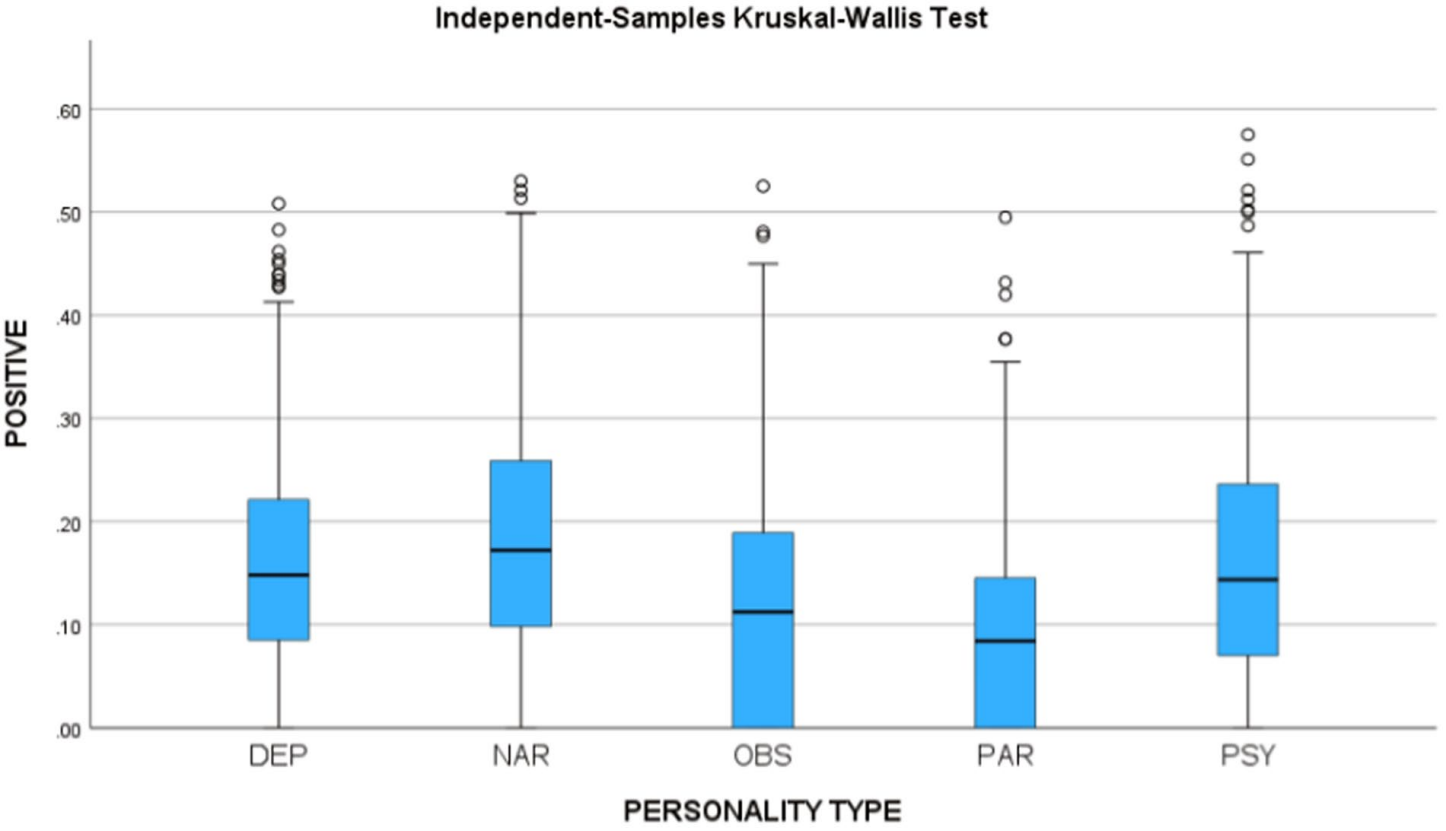
Positive sentiment scores.

Using IBM SPSS for Windows, version 29, we measured the sentiment of the utterances and tested the two hypotheses. Using the Independent-Samples Kruskal-Wallis Test, the differences between the personality types were found statistically significant for Negative sentiment (H = 544.32, p < 0.001) and Positive sentiment (H = 406.66, p < 0.001). Figure 3 presents the median of the negativity scores for the personality types, and Fig. 4 presents the median of the positivity scores for the personality types.

As we can see, the results support the hypotheses, and a post-hoc analysis supports our hypothesis (p < 0.001).

Test 4.3

We used a specifically challenging task for the final test to support the synthetic utterances’ ecological validity. Game of Thrones^23^ is a highly successful TV series based on the fantasy novels by George R. Martin. Some of the characters have a clear personality type. Ramsay Bolton is a clear-cut case of a psychopath (https://en.wikipedia.org/wiki/Ramsay_Bolton). Joffrey Baratheon is the son of Cersei Lannister and her brother Jamie. He is also a *psychopath* with sadistic behavior (https://en.wikipedia.org/wiki/Joffrey_Baratheon). Tyrion (https://en.wikipedia.org/wiki/Tyrion_Lannister) is a dwarf who is blamed for the death of his mother, who died while giving him birth. Suffering from his appearance, a sense of guilt imposed by his family blaming him for the death, and his father’s alienation, he presents *depressive* behavior expressed in heavy drinking and bitter and cynical beliefs about himself and the world. Finally, the *High Sparrow* is a religious leader who shows signs of obsessive behavior.

We hypothesized that if our utterances validly represent personality types, then when using the SetFit model trained on the synthetic utterances, the depressive score of Tyrion should be higher than the others, the anti-social psychopathic score of Ramsay and Joffrey should be higher than the other characters, and the High Sparrow should score higher on the obsessive personality.

**Fig. 5:**
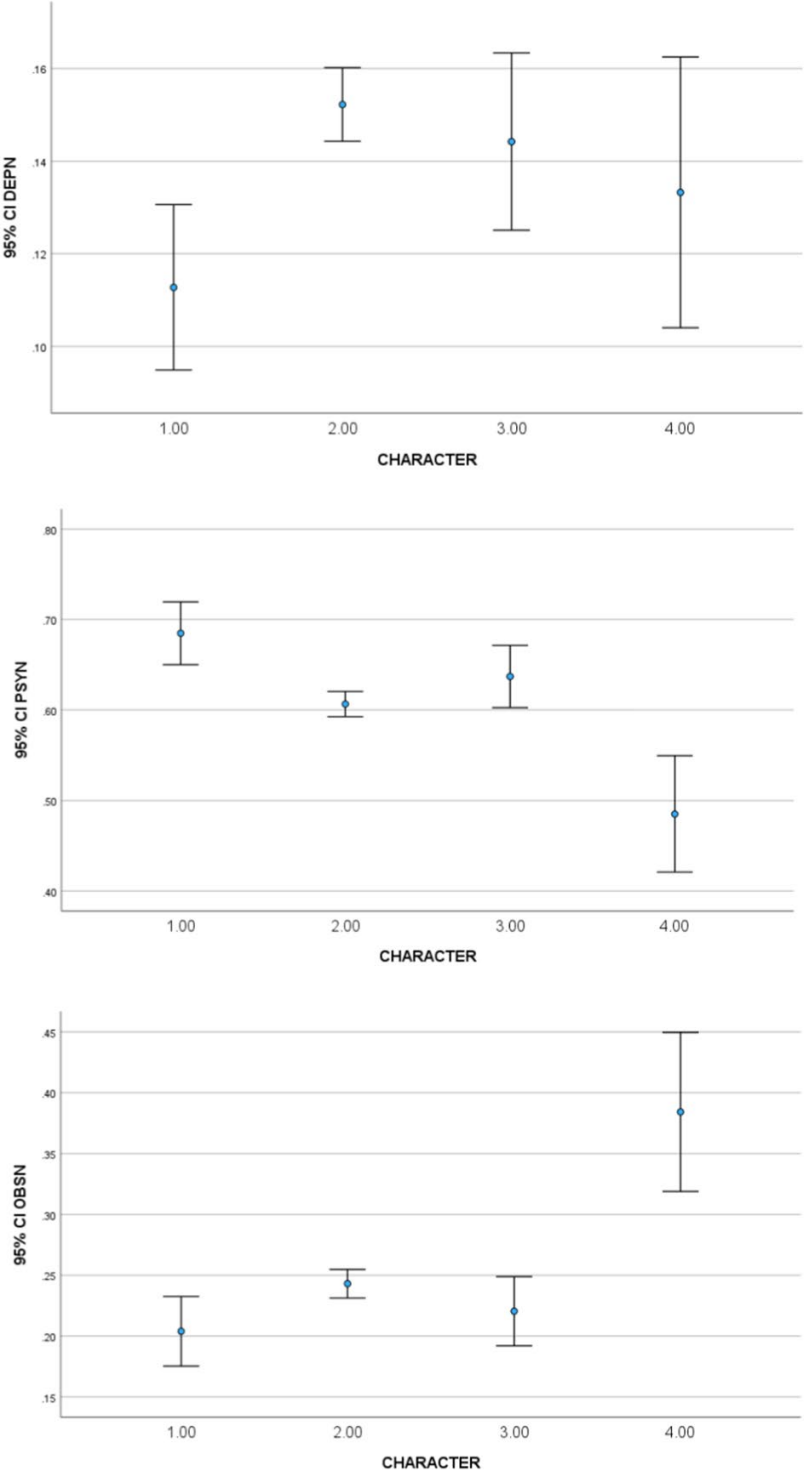
(**a**–**c**) Personality scores of DEP (**a**), OBS (**c**), and PSY (**b**) for the four characters plus 95% Confidence Interval.

Using the script of all seasons^23^, we analyzed all the utterances produced by these four characters. The normalized scores for Joffrey (1), Tyrion (2), Ramsay (3), and the High Sparrow (4) are presented in Fig. 5a–c. The scores were transformed into a 0-1 scale using the MinMax transformation and calculated as follows: PERSONALITY TYPE = PERSONALITY TYPE_1_ – (MEAN (PERSONALITY TYPE_2_, PERSONALITY TYPE_3_)). For example, DEP = DEP- (MEAN (OBS, PSY)). The normalized personality scores (depressivity, obsessional and psychopathic) for each of the four characters are presented in the following figures.

We can see that the scores support our hypotheses. Using one-way ANOVA with character as the independent variable and the personality score as the dependent variable was found to be statistically significant for depressive personality (F (3, 2389) = 14.75, p = 0.003), obsessional personality (F (3, 2389) = 12.59, p < 0.001), and antisocial psychopathic personality F (3, 2389) = 11.97, p < 0.001).

Test 4.4.

We used the results of the LangChain previously presented and the generated textual data concerning the utterances expressed: major theme, beliefs about self/others, and major defense mechanism.

We hypothesized that if the synthetic utterances validly represent the personality types, then (1) The negative sentiment of beliefs about self should be higher for depressive personality, (2) The positive sentiment for beliefs about self should be the highest for narcissistic personality, and (3) The negative sentiment for beliefs about others should be the highest for the paranoid personality. We identified the sentiment in each utterance using IBM SPSS.

**Fig. 6:**
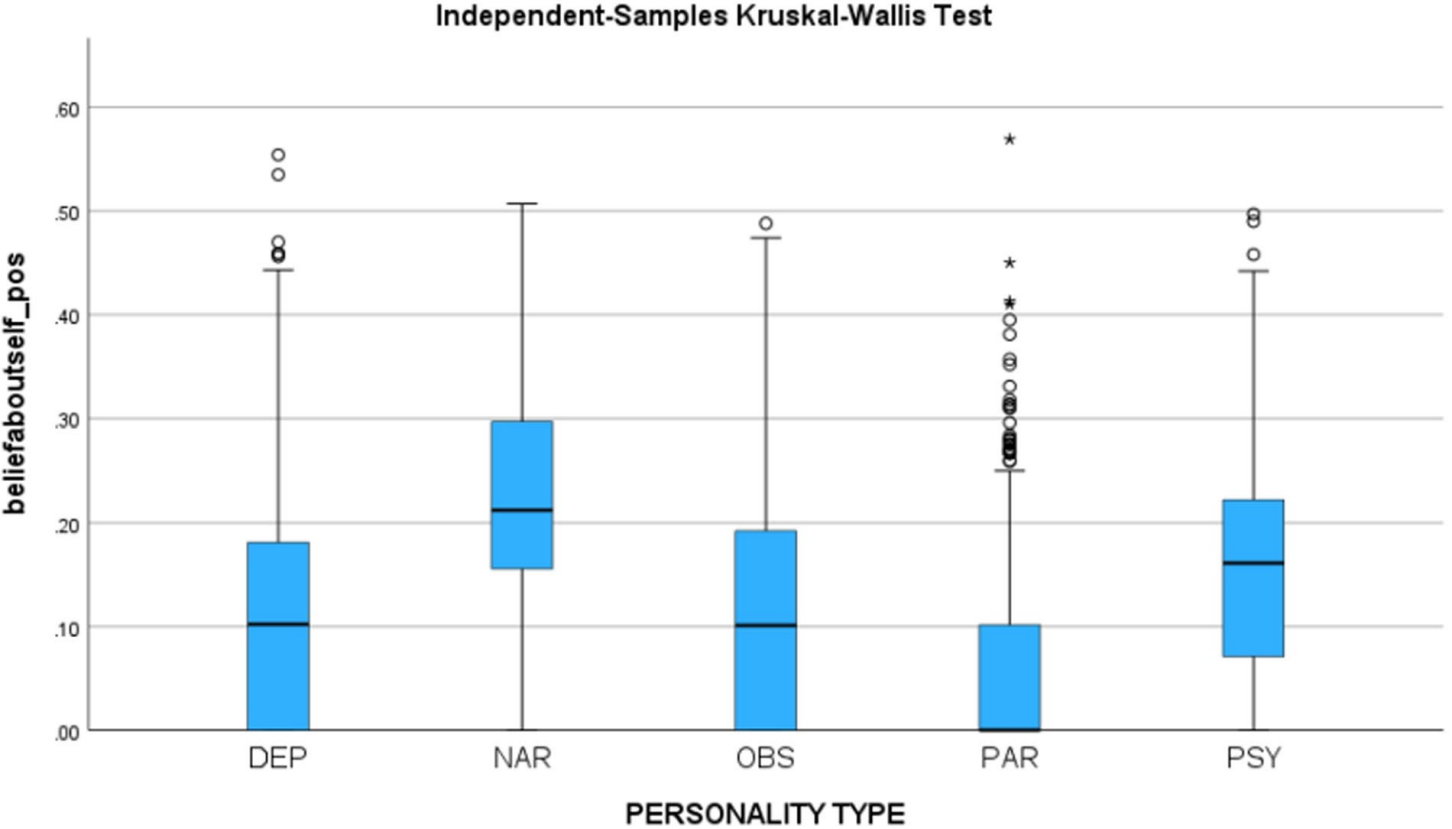
Positive sentiment associated with beliefs about self, for each personality type.

**Fig. 7:**
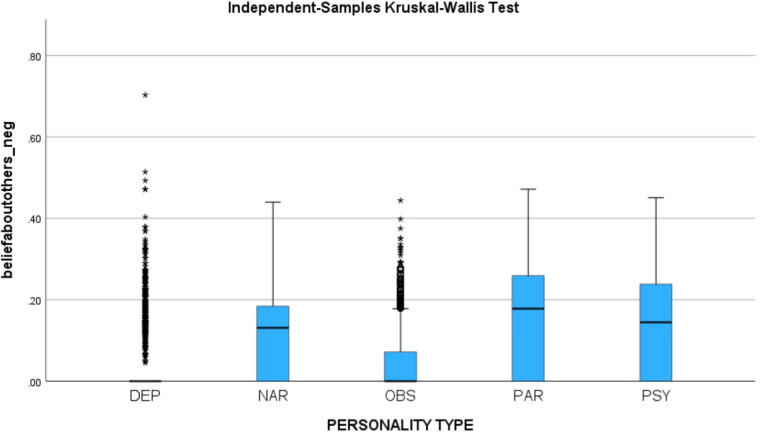
Negative sentiment associated with beliefs about others, for each personality type.

Using the Independent-Samples Kruskal-Wallis Test, a statistically significant difference was found between the personality types for positive sentiment of beliefs about self (H = 1884, p < 0.001) and negative sentiment for beliefs about others (H = 912, p < 0.001). All hypotheses have been confirmed at p < 0.001. See Fig. 6, which shows the positive sentiment associated with beliefs about self for each personality type, and Fig. 7, which shows the negative sentiment associated with beliefs about others for each personality type.

## Data Availability

The Data Records are published in Figshare: Neuman, Y., Cohen, Y. A dataset of personality utterances. *figshare* 10.6084/m9.figshare.24971943.v1. All files are CSV files with a very simple structure. The files are (1) A list of the 100 personas (2) The file with the personality types, utterances, and accompanying data. The link also includes the code for running the SetFit model.
